# Microtomographic Characterization of Morse-Tapered Implant Systems as Material–Biological Interfaces: Implications for Peri-Implant Soft Tissue Stability

**DOI:** 10.3390/ma19153272

**Published:** 2026-08-03

**Authors:** Michele Furlani, Carmen Mortellaro, Alessandra Giuliani

**Affiliations:** 1 Dipartimento di Scienze della Vita, della Salute e delle Professioni Sanitarie, Universita degli Studi “Link Campus University”, 00165 Rome, Italy; m.furlani@unilink.it; 2 Departmental Faculty of Medicine, UniCamillus—Saint Camillus International University of Health Sciences, 00131 Rome, Italy; carmen.mortellaro@unicamillus.org; 3 Dipartimento di Scienze Cliniche Specialistiche e Odontostomatologiche, Universita Politecnica delle Marche, 60131 Ancona, Italy

**Keywords:** Morse-tapered connection, conometric retention, implant–abutment interface, abutment–prosthetic interface, abutment geometry, surface topography, micro-computed tomography, peri-implant soft tissue

## Abstract

**Highlights:**

- Connection geometry, abutment morphology, and surface topography influence peri-implant soft tissue integration.
- Phase-contrast micro-computed tomography enables 3D imaging and quantification of collagen architecture.
- 3D collagen organization around Morse-tapered abutments supports biomechanical hypotheses on mucosal seal stability and force distribution.

**Abstract:**

Advanced dental implant systems are clinically successful when their material properties, surface topography, abutment geometry and implant–abutment connection design support both mechanical stability and biological integration. In Morse-tapered implant–abutment systems and conometric prosthetic retention systems, the precision of conical interfaces has traditionally been discussed mainly in biomechanical terms; however, their clinical performance also depends on the formation and maintenance of a stable peri-implant soft tissue seal. This review examines micro-computed tomography (µCT), with emphasis on synchrotron radiation-based phase-contrast micro-computed tomography (SR-PhC-µCT), as an advanced characterization strategy for evaluating how implant and abutment design influence the three-dimensional architecture of peri-implant connective tissues. The review summarizes the biological organization of the peri-implant mucosa, the technical basis of absorption- and phase-contrast microtomography, sample preparation protocols, segmentation workflows, artificial intelligence-assisted image analysis and quantitative morphometric descriptors of collagen organization. Particular attention is given to human retrieval and biopsy studies of Morse-tapered/conometric systems, where three-dimensional imaging has revealed interwoven circumferential and longitudinal collagen bundles around the transmucosal implant component. These microarchitectural features have been directly visualized by SR-PhC-µCT and histology and may represent a biomechanically plausible basis for mucosal sealing and force distribution. However, their direct relationship with marginal bone preservation and long-term clinical outcomes remains to be demonstrated in longitudinal studies. Within the scope of advanced dental materials, the novelty of this review lies in framing SR-PhC-µCT, correlative histology and AI-assisted segmentation as enabling tools within a materials-design framework for implant–abutment optimization. In this framework, peri-implant collagen architecture is interpreted as an exploratory structural readout of the interaction among connection design, abutment geometry, surface topography and biological response. This approach does not yet establish validated clinical biomarkers, but it may generate testable hypotheses for future studies aimed at improving peri-implant soft tissue stability and implant–abutment system design.

## 1. Introduction

Dental implant systems are advanced biomedical materials whose performance depends on a coordinated interaction among bulk material properties, surface topography, component geometry, prosthetic connection design and host–tissue response. In contemporary implant dentistry, the aesthetic and functional expectations of patients are increasing, while clinicians require implant–abutment assemblies that can withstand repeated mechanical loading in the complex oral environment. Therefore, the evaluation of implant materials cannot be limited to mechanical resistance or osseointegration alone; it must also include the biological behavior of the transmucosal interface, where the implant component interacts with the peri-implant mucosa [1,2,3].

Morse-tapered systems represent a clinically relevant example of this material-design-biological relationship. Their internal tapered connection has been reported to improve mechanical coupling and reduce micromotion and microgap formation at the implant–abutment interface, factors that may limit bacterial leakage under specific experimental conditions. At the same time, the macrogeometry of the abutment, collar profile, surface finish and microtopography can modulate epithelial adaptation, fibroblast activity, collagen deposition and the spatial organization of the supracrestal connective tissue [4,5]. These factors are directly relevant to the design-to-application pathway targeted by advanced dental materials research. In this review, Morse-tapered and Morse cone systems refer primarily to internal conical implant–abutment connections, whereas conometric systems are discussed when the conical friction-fit principle involves prosthetic retention or an abutment–prosthetic cap interface.

The peri-implant mucosal complex, with special reference to supracrestal connective tissue, acts as a protective barrier that is comparable to, but structurally different from, the dentogingival attachment around natural teeth. A stable peri-implant soft tissue seal is considered important for limiting microbial and inflammatory challenge at the transmucosal interface and has been associated with peri-implant tissue stability; however, direct causal links with marginal bone preservation depend on multiple biological, prosthetic and patient-related factors. Conversely, inadequate tissue adaptation, unfavorable component geometry or disruption of the mucosal barrier may predispose the peri-implant environment to inflammation and bone remodeling. Understanding this interface is therefore essential for the rational design of implant materials and prosthetic components.

Conventional histology remains fundamental for evaluating peri-implant soft tissues; however, it is destructive, section-dependent and vulnerable to artifacts associated with fixation, dehydration, embedding and cutting [6]. Electron microscopy provides high spatial resolution but generally examines small regions and does not reconstruct the circumferential continuity of the tissue interface. Micro-computed tomography (µCT), particularly synchrotron radiation-based phase-contrast µCT (SR-PhC-µCT), addresses this limitation by enabling non-destructive three-dimensional (3D) analysis of weakly absorbing collagen-rich tissues [7,8,9,10,11,12]. In this review, microtomography is framed as an advanced characterization method for dental implant materials and abutment designs, with the specific aim of linking implant geometry, connection precision and surface engineering to peri-implant soft tissue architecture and clinical performance.

The main contribution of this review is therefore to propose a materials-design and translational framework for interpreting Morse-tapered and conometric implant systems as material–biological interfaces. Within this framework, SR-PhC-µCT, correlative histology and AI-assisted segmentation are considered enabling characterization tools; peri-implant collagen architecture is considered a measurable biological readout; and implant–abutment connection design, abutment geometry and surface topography are considered modifiable material-design variables. The review does not aim to establish validated clinical biomarkers or causal links with long-term outcomes, but rather to identify structural descriptors and testable hypotheses that may guide future implant–abutment optimization.

## 2. Search Strategy and Scope

This article was designed as a focused narrative review; the literature search was performed to identify studies relevant to the three main themes of the manuscript: (i) peri-implant soft tissue architecture around dental implants, with particular attention to Morse-tapered and conometric implant systems; (ii) the influence of implant–abutment connection design, abutment macrogeometry, surface topography and prosthetic component configuration on peri-implant tissue integration; and (iii) the application of µCT, SR-PhC-µCT and related image-analysis workflows for the 3D characterization of collagen-rich biological tissues.

The search was conducted using PubMed/MEDLINE, Scopus, Web of Science and Google Scholar. The following keywords and combinations were used: “Morse taper implant”, “Morse cone implant”, “conometric implant”, “implant–abutment connection”, “peri-implant soft tissue”, “peri-implant mucosa”, “connective tissue attachment”, “collagen fibers”, “collagen architecture”, “abutment geometry”, “surface topography”, “micro-computed tomography”, “micro-CT”, “synchrotron radiation”, “phase-contrast microtomography”, “SR-PhC-µCT”, “virtual histology”, “semantic segmentation”, “deep learning”, and “U-Net”. Additional relevant articles were identified by screening the reference lists of selected publications.

The search primarily included articles published in English from 1990 to 2025, although older foundational papers were retained when they provided essential methodological or biological background. Eligible publications included narrative and systematic reviews, animal and human histological studies, human biopsy or retrieval studies, technical papers on X-ray microtomography and phase-contrast imaging, and studies applying image analysis or artificial intelligence-based segmentation to collagenous tissues. Emphasis was placed on human retrieval and biopsy studies involving Morse-tapered or conometric implant systems, because these studies provide direct evidence on the 3D organization of peri-implant connective tissue at clinically relevant implant–abutment interfaces.

Studies were excluded when they were not related to dental implants, peri-implant soft tissues, collagen organization, implant–abutment design, or 3D imaging methods. Publications focused exclusively on hard tissue osseointegration, implant survival rates without soft tissue or interface analysis, or purely clinical outcomes without material-design or imaging relevance were not prioritized. Because this review is narrative in nature, no formal meta-analysis, risk-of-bias assessment, or PRISMA-based screening was performed. Instead, the included studies were synthesized thematically to highlight the relationship between implant material design, abutment geometry, microtomographic characterization and peri-implant soft tissue stability.

The key studies most directly supporting the focus of this review are summarized in Table S1. To improve transparency and reproducibility, Table S1 reports, whenever available from the original publications, the number of implants, biopsies or specimens analyzed; the implant or abutment system; abutment macrogeometry; surface characteristics or treatment; healing time; loading condition; imaging modality; voxel size or spatial resolution; main structural or morphometric outcomes; and key limitations. This table also clarifies the rationale for emphasizing selected human retrieval and biopsy studies on Morse-tapered and conometric implant systems, while distinguishing these from broader methodological, technical and background references used to support the discussion.

For terminology consistency, this review uses “Morse-tapered” and “Morse cone” to refer to internal conical implant–abutment connections, whereas “conometric” refers to conical friction-fit retention principles, particularly at the abutment–prosthetic cap interface. These interfaces are considered separately because their mechanical precision, microgap formation, bacterial leakage potential, prosthetic stability and peri-implant tissue implications are not necessarily equivalent.

## 3. Biological Architecture of the Peri-Implant–Soft Tissue Interface

### 3.1. The Peri-Implant Mucosal Seal

The soft tissues surrounding the transmucosal implant component are commonly referred to as peri-implant mucosa [13]. Their protective function depends on the establishment of an epithelial attachment and a connective tissue zone able to withstand microbial, inflammatory and mechanical challenges. A mature connective tissue barrier is considered important for limiting apical inflammatory cell migration and may contribute to the preservation of peri-implant crestal bone.

Animal studies have shown that the vascular supply of peri-implant mucosa differs from that of periodontal tissues. Whereas gingiva around natural teeth receives blood supply both from supraperiosteal vessels and from the periodontal ligament, the peri-implant mucosa is mainly supplied by terminal branches originating from periosteal vessels [14]. Morphometric analyses in dogs further described a connective tissue region composed of an inner, vessel-poor zone adjacent to the implant surface and a more peripheral zone containing collagen fibers, fibroblasts and vascular structures [15].

### 3.2. Human Connective Tissue Organization Around Implants

Human biopsy and retrieval studies indicate that the connective tissue barrier around implants is a specialized structure characterized by high collagen content and relatively low cellular and vascular fractions [16,17,18,19,20]. This peri-implant connective tissue interface differs substantially from the periodontal ligament. Around natural teeth, collagen fibers insert into cementum and alveolar bone through a functionally organized attachment apparatus, including Sharpey fibers. Around dental implants, by contrast, collagen fibers are generally described as running parallel, obliquely or circumferentially to the implant or abutment surface, while true perpendicular or radial insertion into titanium has been reported only occasionally and inconsistently [21,22,23,24,25,26]. The peri-implant connective tissue interface is therefore generally considered a collagenous adaptation rather than a periodontal-like insertion.

This arrangement has been interpreted as a dense collagenous cuff that could contribute to mechanical stabilization and biological sealing. However, direct functional evidence linking this specific fiber organization to measurable sealing capacity or clinical outcomes remains limited. The matrix is predominantly composed of fibrillar collagen, especially types I and III, whose organization contributes to tissue tensile properties and influences cell behavior [6,27]. Collagen architecture also provides topographical and mechanical cues for fibroblast migration, matrix remodeling and wound healing. Because collagen remodeling is sensitive to mechanical loading and local inflammation, three-dimensional analysis of collagen-rich peri-implant tissue is important for understanding both physiological healing and pathological breakdown [28,29,30].

The SR-PhC-µCT studies reviewed here are consistent with previous histological and ultrastructural evidence but extend it by providing volumetric visualization of peri-implant collagen organization. These studies show that collagen bundles around transmucosal implant components may be arranged as circumferential/transverse and longitudinal networks, while radial insertion into the implant surface is generally not observed. Thus, SR-PhC-µCT does not replace conventional histology but complements it by allowing peri-implant collagen architecture to be examined as a 3D structural network rather than only through selected two-dimensional section planes.

### 3.3. Why Three-Dimensional Analysis Is Required

The main limitation of conventional histology is that it reduces a circumferential and spatially heterogeneous interface to selected two-dimensional planes. Fiber populations that appear parallel, oblique or transverse in one section may be part of a more complex three-dimensional network. Consequently, volumetric techniques are needed to evaluate continuity, interconnection, anisotropy and spatial gradients within this and similar connective tissue structures [31,32,33,34]. In particular, SR-PhC-µCT has recently allowed investigators to distinguish circular and longitudinal collagen bundles around implant abutments, identify sparse vascular structures and compare three-dimensional observations with light microscopy [8,9,10].

Taken together, these biological considerations provide the conceptual basis for the following sections of this review. The peri-implant mucosal seal and the three-dimensional organization of collagen-rich connective tissue are not discussed here as isolated biological phenomena, but as structural features that can be visualized, quantified and interpreted in relation to implant–abutment design. Accordingly, the subsequent sections examine how SR-PhC-µCT, segmentation workflows and morphometric descriptors can translate this biological architecture into measurable parameters relevant to advanced dental implant material design.

## 4. Microtomographic Characterization Methods for Dental Implant Materials and Peri-Implant Tissues

### 4.1. Absorption-Based µCT

X-ray tomography generates a virtual three-dimensional representation of a specimen by acquiring radiographic projections over multiple angular positions and reconstructing cross-sectional slices through mathematical algorithms based on the inverse Radon transform [7,35,36]. In absorption-based CT, image contrast reflects the attenuation of X-rays according to material composition, thickness and density. Dense mineralized tissues are therefore readily visualized, whereas soft tissues with similar attenuation coefficients are more difficult to separate.

MicroCT extends this approach to micrometric and submicrometric length scales. It is widely used to quantify morphometric parameters, including volume fraction, thickness, spacing, connectivity and anisotropy. For peri-implant research, these outputs are attractive because they allow three-dimensional evaluation of tissue adaptation and structural organization. However, conventional laboratory µCT often provides insufficient contrast for unstained connective tissues unless staining, contrast agents or phase-contrast strategies are introduced.

### 4.2. Advantages of Synchrotron Radiation-Based µCT

Synchrotron radiation offers high brilliance, coherence and collimation, enabling quasi-monochromatic imaging and precise control of photon energy [37]. Compared with polychromatic laboratory sources, monochromatic beams reduce beam-hardening artifacts, improve signal-to-noise ratio and support quantitative density-based analyses [38,39,40,41,42,43]. High flux allows shorter exposure times, high-resolution datasets and, in some contexts, time-resolved or multiscale imaging [44,45,46].

The coherence of synchrotron radiation is especially relevant for phase-contrast methods. Phase-contrast imaging enhances the detectability of structures with low absorption contrast by exploiting refraction-induced phase shifts rather than attenuation alone [47,48,49,50]. This makes SR-PhC-µCT particularly suitable for resin-embedded peri-implant soft tissues, where collagen bundles and interstitial spaces may have only subtle density differences.

### 4.3. Propagation-Based Phase-Contrast Imaging and Phase Retrieval

**Figure 1:**
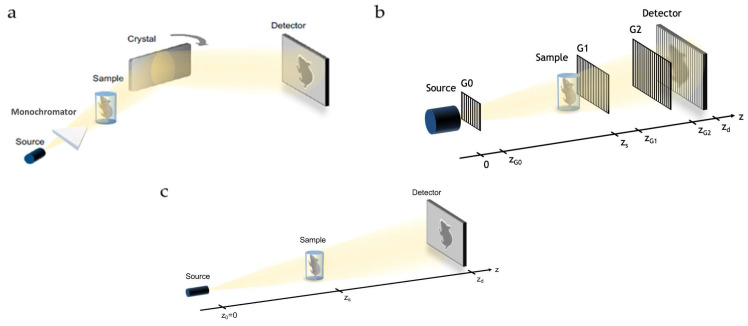
Schematics of the most common phase-contrast setups: (**a**) analyzer-based, (**b**) grating interferometry, and (**c**) propagation-based (Ref. [53], CC-BY 4.0).

Several phase-contrast strategies are available, including free-space propagation, grating interferometry, dark-field imaging, edge illumination and analyzer-based imaging (Figure 1) [47,51,52,53,54,55,56,57,58,59,60]. In peri-implant soft tissue studies, propagation-based imaging is particularly useful because it can be implemented without additional optical elements. When the X-ray beam propagates after passing through the sample, refraction produces interference fringes that enhance edges between materials with different refractive indices.

Phase-retrieval algorithms can convert edge-enhanced projections into images with improved area contrast. Paganin’s single-distance method is widely used because it is robust, noise-suppressing and suitable for specimens that can be approximated by a homogeneous ratio between the real and imaginary components of the refractive index [61,62]. Critical acquisition parameters include photon energy, sample-to-detector distance, voxel size, detector performance, specimen dimension and composition [63]. In practice, voxel size determines field of view and sampling, whereas true spatial resolution is also influenced by source size, detector point-spread function and reconstruction workflow. For collagen-rich peri-implant soft tissues, the selection of acquisition parameters reflects a balance between contrast, spatial resolution, field of view and radiation dose. Photon energy must be sufficient to penetrate resin-embedded specimens while preserving phase sensitivity at collagen–matrix interfaces. The sample-to-detector distance controls the development of propagation-based phase-contrast fringes, which enhance the visibility of weakly absorbing collagen bundles. Exposure time and the number of projections influence signal-to-noise ratio and reconstruction quality, whereas angular range and angular step affect spatial sampling and artifact reduction. Therefore, these parameters are reported together with voxel size to allow comparison across studies.

### 4.4. Preservation of Sample Integrity and Virtual Histology

A major advantage of SR-PhC-µCT is the preservation of specimen integrity. The same embedded biopsy can be imaged volumetrically, virtually sectioned in any plane and subsequently guided toward targeted histological sectioning [64,65]. This correlative strategy is particularly valuable in peri-implant research because the soft tissue–implant interface is fragile and easily distorted by mechanical processing. When combined with polarized light microscopy and conventional histology, SR-PhC-µCT provides a bridge between high-resolution two-dimensional validation and three-dimensional microstructural interpretation.

## 5. Practical Workflow for Three-Dimensional Characterization of Implant–Tissue Interfaces

### 5.1. Sample Preparation

Human peri-implant biopsy specimens generally include the abutment and surrounding soft tissues. Samples are fixed in buffered formalin, dehydrated through graded ethanol and embedded in glycolmethacrylate or methyl methacrylate resin. After polymerization, the embedded abutment may be sectioned longitudinally into two portions: one for histological processing and one for microtomographic imaging after careful removal of the metal component [10,66,67].

This preparation is designed to preserve the soft tissue architecture originally surrounding the abutment. The metal component should be thin and free of undercuts to minimize the risk of tearing the interface during removal. Stereomicroscopic control and cautious detachment along the tissue-abutment interface are recommended.

Sample preparation is a critical step because fixation, graded dehydration, resin embedding, sectioning and removal of metallic components may influence tissue morphology, collagen bundle spacing and the preservation of the implant–soft tissue interface. These steps are therefore not merely technical procedures, but potential sources of preparation-related artifacts that must be considered when interpreting SR-PhC-µCT, histological and polarized light microscopy findings.

The workflow also enables transverse and longitudinal histological and polarized light microscopy analyses on selected sections, allowing independent correlative assessment of collagen orientation. In the reviewed studies, histological processing generally involved resin-embedded peri-implant soft tissue specimens sectioned in longitudinal and/or transverse planes, followed by conventional staining such as toluidine blue and acid fuchsin or hematoxylin and eosin, depending on the original protocol. Polarized light microscopy was used to exploit collagen birefringence and to distinguish collagen bundles according to their orientation relative to the section plane and light propagation direction. However, section thickness, staining protocol, microscope configuration and polarization settings were not uniformly reported across all studies and should be standardized in future investigations.

### 5.2. Acquisition, Reconstruction and Software Tools

Reported peri-implant studies have used synchrotron beamlines such as SYRMEP at Elettra, with average or peak photon energies of approximately 17–19.3 keV, exposure times of 0.2–0.4 s per projection, 1800 projections over 180° or 3600 projections over 360°, a sample-to-detector distance of 100 mm and a pixel size of 890 nm, depending on the original study. The experiments were performed using propagation-based phase-contrast imaging, where image contrast is governed by the complex refractive index, defined as *n* = 1 − *δ* + *iβ*, with *δ* describing the real refractive decrement and *β* representing the imaginary component associated with X-ray absorption. Reconstruction has been performed with dedicated tools such as the SYRMEP Tomo Project, while phase retrieval has commonly used Paganin’s method with a fixed δ/β ratio of 100 [61,68].

After reconstruction, 3D datasets can be processed in commercial or open-source platforms. Dragonfly has been used for visualization, machine-learning-assisted segmentation and semantic classification [69]. Fiji/ImageJ (v1.54r), with plugins such as BoneJ (v1.4.3), supports thresholding, preprocessing and morphometric analysis [70,71]. A combined software pipeline is often advantageous: one platform may provide robust AI-assisted labeling, whereas another offers validated morphometric indices.

### 5.3. Segmentation Strategies

Two complementary segmentation approaches are commonly used. Histogram-based Otsu thresholding separates grayscale classes by maximizing inter-class variance and is useful when intensity differences are sufficiently stable. However, collagen bundles with different orientations may have similar gray levels, making thresholding insufficient for orientation-specific classification.

Semantic segmentation overcomes this limitation by assigning class labels based on grayscale, texture, shape and contextual features. U-Net architectures are particularly suited to biomedical images because their encoder–decoder structure combines contextual feature extraction with accurate localization [72,73]. In peri-implant datasets, manually segmented slices can be used to train a three-class model distinguishing background, longitudinal collagen bundles and transverse/circular collagen bundles. Data augmentation, including rotations, flipping, scaling, shearing, cropping and contrast adjustments, helps increase training variability and reduce overfitting.

In the peri-implant soft tissue study applying U-Net-based semantic segmentation, the network was trained to distinguish three classes: background, transverse/circular collagen bundles and longitudinal collagen bundles [73]. The training dataset consisted of 50 manually segmented slices, obtained from selected volumes of two samples, and the software internally separated the dataset into training and validation subsets using a 90/10 split. Data augmentation was applied to increase variability and reduce overfitting. The study reported categorical accuracy during training and validation, but did not provide Dice coefficient, intersection over union, precision or recall values. Therefore, segmentation-derived morphometric comparisons should be interpreted in light of this validation limitation.

Because semantic segmentation directly affects the reliability of subsequent morphometric measurements, validation should be reported in detail. Future studies should specify the number of manually annotated slices or volumes used for model training, the number and expertise of annotators, and whether annotations were performed independently or by consensus. Interobserver agreement should be assessed whenever multiple annotators are involved. The training, validation and test split should also be clearly described, and segmentation performance should be quantified using standard metrics such as the Dice similarity coefficient, intersection over union, precision, recall, sensitivity, specificity and class-specific accuracy. These metrics should be reported separately for each semantic class, particularly when distinguishing transverse/circular and longitudinal collagen bundles. This is especially relevant because collagen bundles with different orientations may show similar grayscale values and may differ mainly in texture, shape and spatial context.

Segmentation performance should also be evaluated across different tissue maturation stages and clinical conditions. Immature connective tissue may show lower collagen density, less defined bundle orientation and greater morphometric variability than mature peri-implant connective tissue, potentially reducing classification accuracy. Whenever possible, AI-based segmentation should be validated against independent reference methods, including histology and polarized light microscopy, to confirm collagen orientation and tissue compartment identification. For this validation to be reproducible, future studies should report the thickness of histological sections, the staining method, the number and orientation of analyzed sections, microscope magnification, polarization configuration and the criteria used to assign collagen bundles to transverse/circular or longitudinal categories. Without such validation, morphometric differences derived from segmented datasets should be interpreted cautiously.

### 5.4. Quantitative Morphometric Descriptors

Morphometric analysis converts qualitative impressions of tissue organization into measurable structural descriptors within a selected region of interest (ROI). In the reviewed studies, these parameters were derived from segmented 3D datasets using image-analysis workflows adapted from trabecular bone and fibrous network morphometry. Collagen volume fraction (Vol.%) was defined as the ratio between the segmented collagen bundle volume and the total analyzed volume, according to the relationship Vol.% = CollV/TV × 100, where CollV is the segmented collagen volume and TV is the total ROI volume. Bundle thickness (Th), bundle number (Nr) and bundle spacing (Sp) describe the local packing of collagen structures and are derived from 3D thickness and spacing algorithms commonly used in morphometric analysis. Bundle thickness estimates the mean local thickness of segmented collagen structures, bundle number estimates the number of collagen bundles per unit length, and bundle spacing estimates the mean distance between adjacent segmented collagen structures. The degree of anisotropy (DA) is a dimensionless index describing the extent of preferential orientation within the segmented structure. Because DA calculation and interpretation may depend on the software implementation and convention used, DA values should always be interpreted according to the definition adopted in the original study and in conjunction with visual, histological or polarized light microscopy validation. Connectivity density (Conn.D) estimates the interconnection of the collagen network per unit volume or per voxel-based unit, depending on the original study. Fractal dimension (FD), often calculated using box-counting approaches, describes the structural complexity of the segmented collagen network across spatial scales [71,73].

Preprocessing influences the reliability of these measurements. Median filtering, edge detection and histogram equalization may be applied before anisotropy and connectivity analyses, while more conservative filtering may be used for volume-fraction measurements. Standardized region-of-interest selection is essential because peri-implant connective tissue shows spatial gradients from the abutment interface toward peripheral mucosa.

The biological interpretation of these morphometric descriptors requires caution. Collagen volume fraction, bundle thickness, number and spacing describe the amount and spatial packing of collagen structures within a selected region of interest, but they do not directly measure sealing capacity, mechanical resistance or tissue health. Similarly, connectivity density reflects the degree of interconnection within the segmented collagen network; however, higher connectivity should not automatically be interpreted as a more effective biological seal unless supported by functional or longitudinal clinical evidence.

The degree of anisotropy provides information on preferential fiber alignment, but its meaning is context dependent. Higher anisotropy may indicate tissue maturation, mechanical adaptation to directional loading or alignment imposed by local abutment geometry. Conversely, lower anisotropy may reflect a more isotropic network, immature tissue organization, regional heterogeneity or segmentation uncertainty. Therefore, anisotropy should be interpreted together with histological validation, tissue maturation stage, loading condition, abutment design and region-of-interest selection.

The distinction between transverse/circular and longitudinal collagen bundles is useful for describing three-dimensional architecture, but these fiber populations should not be assumed to have fixed or mutually exclusive biological functions. Transverse or circumferential bundles may be consistent with a sealing or encircling organization, whereas longitudinal bundles may be consistent with axial force transmission; however, these interpretations remain biomechanically plausible hypotheses rather than directly demonstrated functional roles. Moreover, morphometric values may be influenced by voxel size, true spatial resolution, phase-retrieval parameters, reconstruction algorithms, preprocessing filters, thresholding or AI-based segmentation strategies. Standardized acquisition, segmentation and validation workflows are therefore required before these parameters can be compared across studies or considered reproducible biomarkers of peri-implant tissue performance.

When morphometric data are summarized in this review, they are reported as descriptive values extracted or adapted from the original publications. No additional statistical testing was performed on these data for the present review. Therefore, any statistical significance, *p* value or multiple-comparison correction should be interpreted according to the methods and assumptions of the original study from which the data were derived.

## 6. Human Evidence Linking Implant–Abutment Design to Peri-Implant Soft Tissue Architecture

The healing times and loading conditions reported in the following studies were not selected as part of a new experimental design for the present review but were extracted from the original human retrieval and biopsy studies. These time points reflect the clinical protocols, retrieval indications and ethical constraints of each study. Therefore, comparisons between early healing, delayed healing, loaded and non-loaded conditions should be interpreted descriptively rather than as evidence from a controlled longitudinal time-series analysis. Most available peri-implant SR-PhC-µCT observations refer to early or intermediate healing phases, including 4–8 weeks, 45 days or 3 months after implant or abutment placement, depending on the original study design. These intervals are useful for investigating early collagen organization and connective tissue adaptation, but they cannot define the full maturation kinetics of the peri-implant mucosal seal.

Moreover, it should be emphasized that SR-PhC-µCT, histology and polarized light microscopy directly demonstrate the presence, orientation and spatial interconnection of collagen bundles. By contrast, the contribution of these architectures to mucosal sealing, load dissipation, marginal bone preservation or long-term implant stability should currently be regarded as a biomechanically plausible interpretation rather than as a clinically proven effect.

### 6.1. Interwoven Collagen Network Around Morse-Tapered Abutments

A human retrieval study of three Morse-tapered implants subjected to immediate functional loading and retrieved after 4–6 weeks provided one of the first three-dimensional descriptions of peri-implant connective tissue architecture by SR-PhC-µCT [10]. Histology showed a junctional epithelium in close apposition to the implant surface, dense connective tissue and no inflammatory infiltrate. Polarized light microscopy identified collagen bundles oriented both parallel to the implant–abutment axis and circumferentially around the abutment.

**Figure 2:**
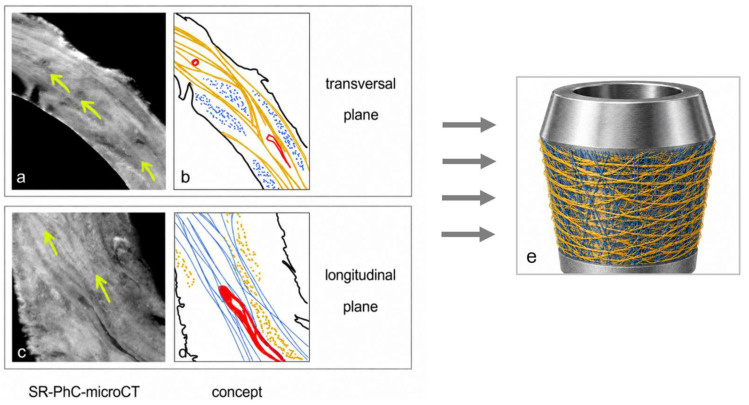
SR-PhC-µCT-based visualization and conceptual interpretation of peri-implant connective tissue architecture around a Morse-tapered abutment. (**a**,**c**) Representative two-dimensional SR-PhC-µCT slices acquired in the transverse (**a**) and longitudinal (**c**) planes, showing the connective tissue bundles (green arrows) surrounding the transmucosal implant component. (**b**,**d**) Schematic interpretation of the structures identified in the corresponding transverse (**b**) and longitudinal (**d**) planes; circumferential/transverse collagen bundles are shown in yellow, longitudinal collagen bundles in blue and sparse vascular structures in red. (**e**) Conceptual 3D rendering of the proposed interwoven collagen organization. Panel e is not a direct SR-PhC-µCT reconstruction or quantitative segmentation output, but a conceptual visualization generated by ChatGPT-5.6 Thinking (OpenAI) under author instruction and supervision to illustrate the spatial relationship between circumferential and longitudinal collagen bundles. Panels a–d are modified from Iezzi et al. [10] under a CC BY 4.0 license.

The 3D SR-PhC-µCT reconstructions refined this interpretation by showing a complex interwoven scaffold composed of longitudinal collagen bundles and circular/transverse bundles (Figure 2). Circular bundles were not limited to the outer connective tissue region; rather, they contributed substantially to the connective tissue matrix and interacted with longitudinal elements. No clear radial insertion into the implant surface was detected. This architecture suggests that the peri-implant connective tissue cuff may function as a spatially integrated mesh rather than as a random or poorly organized bundle arrangement. It should be emphasized that SR-PhC-µCT and histology directly demonstrate the presence, orientation and spatial interconnection of collagen bundles, whereas their role in mucosal sealing, load dissipation or marginal bone protection should currently be regarded as a biomechanically plausible interpretation rather than a clinically proven effect.

A regional gradient was also observed. Fibers closer to the abutment tended to be more regular and aligned, while peripheral regions showed greater heterogeneity. Quantitative morphometry provided representative structural descriptors of collagen organization in three human retrieved implants. The analyzed regions of interest included transverse/circular collagen bundles, inner longitudinal collagen bundles and outer longitudinal collagen bundles. These values, reported as mean ± standard deviation, suggested comparable collagen volume and structural complexity between circular and inner longitudinal compartments, whereas the outer longitudinal region showed thicker bundles and reduced spacing (Table 1). Because of the small sample size and the use of dataset-specific metrics, these values should be interpreted descriptively rather than as generalizable reference ranges.

### 6.2. Effects of Abutment Macrogeometry and Surface Microtopography

A human observational case series investigated soft tissue maturation around three healing abutment designs in Morse cone implant systems: convergent abutments with an ultrathin threaded microsurface, machined convergent abutments and machined divergent abutments [66]. After three months, biopsies from 22 analyzable specimens were evaluated by histology, polarized light microscopy and SR-PhC-µCT.

All groups showed healthy peri-implant mucosa with a defined junctional epithelium and no inflammatory infiltrate. The connective tissue component, however, differed according to abutment design. Convergent configurations were associated with greater connective tissue height than divergent abutments. Collagen orientation also followed the abutment profile: divergent abutments tended to show horizontal-to-oblique bundles, whereas convergent abutments showed fibers mainly aligned with the long axis of the implant–abutment complex.

3D SR-PhC-µCT analysis showed that convergent abutments, particularly those with an ultrathin threaded microsurface, were associated with higher collagen volume fraction and connectivity density than divergent machined abutments in selected comparisons. These results suggest that abutment macrogeometry and microtopography may be associated with measurable differences in collagen deposition and spatial interconnection within the soft tissue cuff. However, because no validated biological or clinical threshold exists for collagen volume fraction or connectivity density, these differences should be interpreted as structural morphometric variations rather than as direct evidence of improved mucosal sealing or long-term clinical performance.

### 6.3. Healing Time, Loading and Force-Related Collagen Organization

Deep-learning-based segmentation has been applied to peri-implant soft tissue specimens collected under different healing and loading conditions [73]. Samples included immediately loaded and non-loaded cases, short-term and long-term healing conditions and different posterior jaw sites (Table 2). Semantic segmentation made it possible to quantify transverse and longitudinal collagen bundles separately, an analysis that thresholding alone could not reliably perform. However, the reliability of these orientation-specific morphometric measurements depends on the quality of manual annotations, the robustness of model validation and the degree of agreement with independent histological or polarized light microscopy findings.

**Figure 3:**
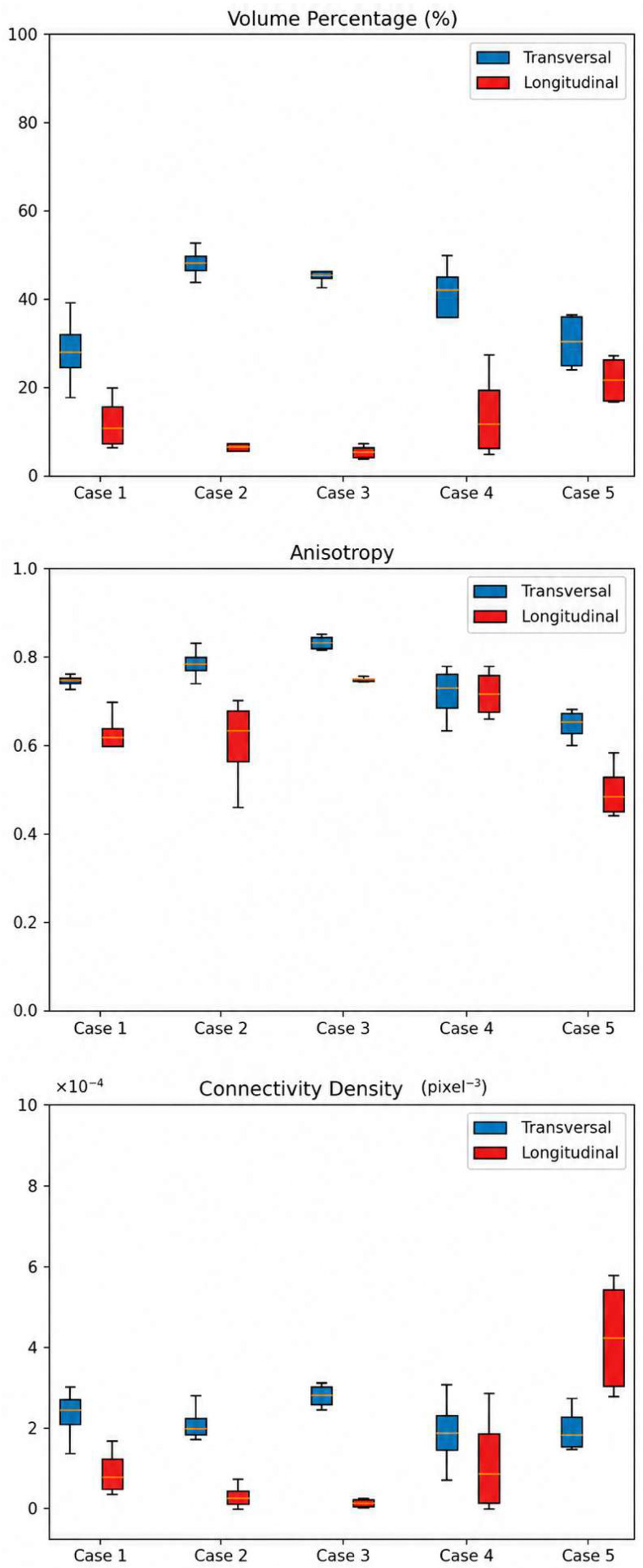
Quantitative morphometric analysis of collagen bundle after deep learning-based semantic segmentation. Box plots showing collagen volume percentage, degree of anisotropy and connectivity density calculated separately for transverse/circular and longitudinal collagen bundles in SR-PhC-µCT datasets. For each sample, four connective tissue subvolumes of 600 × 300 × 300 pixels^3^, corresponding to approximately 0.04 mm^3^, were extracted from the peri-implant soft tissue region at the implant interface and analyzed after U-Net-based semantic segmentation. Transverse/circular collagen bundles are shown in blue and longitudinal collagen bundles in red. Connectivity density is reported in pixel-based units and should be interpreted as a dataset-specific structural descriptor. Adapted from Riberti et al. [73], under a CC BY 4.0 license.

Across most specimens, transverse bundles showed higher volume fraction and connectivity density than longitudinal bundles, whereas longitudinal bundles generally displayed lower anisotropy (Figure 3). These findings indicate direction-dependent collagen organization, with a more interconnected circumferential/transverse component and a distinct longitudinal component. The translation of these descriptors into functional concepts such as load transfer, force dissipation or tissue stabilization remains hypothetical and requires direct biomechanical validation.

The longer-term non-loaded sample (Case 2) was also included in the AI-assisted segmentation study, but it was not part of a planned longitudinal time-series analysis. Therefore, it should be interpreted as a descriptive comparator rather than as evidence of progressive tissue maturation over time.

The exception was immature connective tissue, where collagen organization was less defined and morphometric variability was higher. These data support the hypothesis that collagen microarchitecture may reflect tissue maturation and local biomechanical conditions during peri-implant healing, but they do not directly demonstrate mechanical force pathways.

### 6.4. Retrieved Conometric Implant System: Distinction Between Implant–Abutment Precision and Prosthetic Cap Retention

A clinical case report of a retrieved implant restored with a conometric abutment–prosthetic cap connection further integrated static µCT of the implant–abutment interface with SR-PhC-µCT of surrounding soft tissues [74]. The implant had been removed because of prosthetic mispositioning rather than biological failure. Conventional µCT showed no detectable macro or microgap at the implant–abutment interface in this retrieved case, supporting the mechanical precision of that specific interface under the analyzed conditions [74]. However, this observation should not be generalized to all conometric or Morse-tapered systems without comparative studies.

**Figure 4:**
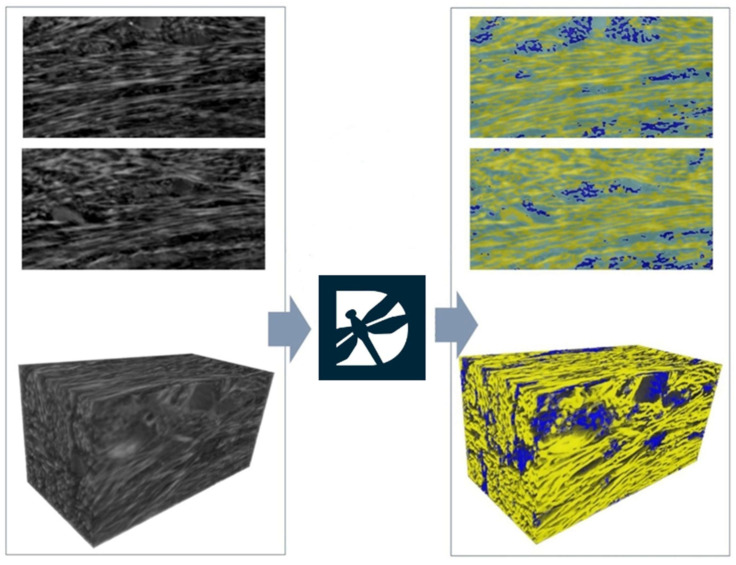
AI-assisted semantic segmentation of peri-implant connective tissue in SR-PhC-µCT datasets. Left panels show representative raw axial slices and the corresponding three-dimensional rendering of a selected subvolume after phase-retrieval tomographic reconstruction. Right panels show the semantic segmentation of the same slices and the related 3D rendering generated using a U-Net-based deep learning approach. The segmentation classified the dataset into three semantic classes: background, transverse/circular collagen bundles and longitudinal collagen bundles. In the segmented images, transverse/circular collagen bundles are displayed in yellow, longitudinal collagen bundles in blue and background in teal. Image processing was performed using Dragonfly software (version 2022.1; Object Research Systems, Montreal, QC, Canada). Segmentation reliability depends on the quality of manual annotations, training/validation strategy and validation against independent reference methods such as histology or polarized light microscopy. From Riberti et al. [74] under a CC BY 4.0 license.

SR-PhC-µCT and AI-assisted segmentation revealed region-specific collagen organization (Figure 4). Transverse bundles predominated and exhibited higher connectivity density, whereas longitudinal bundles showed greater anisotropy, reflecting higher alignment but lower interconnection. Histology confirmed the presence of well-adapted epithelial and connective tissue components, with no visible interfacial gap. These observations support a dual interpretation: under the specific conditions of the analyzed case, the implant–abutment interface showed high mechanical precision, while the surrounding soft tissue exhibited a multidirectional collagen network that may be biomechanically consistent with sealing and load-distribution functions. However, these findings remain exploratory and should not be generalized to all Morse-tapered or conometric systems without comparative studies.

## 7. Implications for Advanced Dental Implant Material Design and Clinical Application

The purpose of this section is to translate the biological and microtomographic observations reviewed above into a materials-design perspective. Accordingly, the following considerations should be understood as a framework for hypothesis generation and preclinical optimization, rather than as evidence of validated clinical biomarkers or established causal pathways.

The evidence reviewed supports the interpretation of Morse-tapered and conometric systems as clinically relevant examples of coupled material–biological interfaces. However, they should not be considered the only implant–abutment configurations capable of supporting organized peri-implant connective tissue architecture, since similar biological responses may also occur around other connection designs, abutment materials and surface modifications.

At the component level, connection precision, alloy behavior, surface finish and abutment macrogeometry contribute to shaping the mechanical environment at the transmucosal interface. At the tissue level, these design features may influence epithelial attachment, fibroblast behavior and collagen maturation. At the microarchitectural level, imaging studies have shown that collagen bundles may form interwoven circumferential and longitudinal networks. From a biomechanical perspective, this organization could support mucosal seal stability and functional force distribution, but these functional implications remain inferential and require direct experimental, computational and clinical validation.

For advanced dental materials research, these observations are relevant because they provide measurable structural descriptors of the peri-implant–soft tissue interface. Circumferential/transverse collagen bundles can be directly identified as partially encircling the abutment, while longitudinal bundles can be distinguished according to their orientation along the implant axis. It is biomechanically plausible that these different fiber populations contribute differently to tissue stability and force distribution; however, direct evidence linking these morphometric features to sealing efficacy, marginal bone preservation or implant survival is not yet available. Parameters such as collagen-specific volume, degree of anisotropy, connectivity density and connective tissue height should therefore be considered exploratory structural descriptors rather than validated clinical biomarkers. Their functional significance for mucosal sealing, force dissipation, marginal bone preservation or implant survival remains to be established through standardized imaging protocols, validated segmentation workflows, biomechanical testing and longitudinal clinical correlation.

Abutment design may influence this architecture. Convergent profiles have been associated with increased vertical dimensions of connective tissue and axial fiber organization, whereas microthreaded or microstructured surfaces have been associated with greater collagen deposition and interconnection in selected studies. These findings align with broader evidence that collagen-based matrices, scaffold properties and local biomechanical stimuli influence peri-implant wound healing and extracellular matrix maturation [75,76,77,78]. Thus, SR-PhC-µCT can support the preclinical and translational optimization of implant–abutment systems by relating design variables to measurable microstructural features of the peri-implant–soft tissue interface.

## 8. Limitations and Future Directions

The evidence discussed in this review is not based solely on in vitro or animal models. The core SR-PhC-µCT evidence is mainly derived from ex vivo human retrieval and peri-implant biopsy studies, while animal, in vitro and broader methodological studies were used as the supporting background literature. Nevertheless, the available human evidence remains limited in sample size, study heterogeneity and independent replication, which restricts the generalizability of the conclusions.

Indeed, although SR-PhC-µCT provides unique 3D information on peri-implant soft tissue architecture, the current evidence remains limited by small sample sizes, heterogeneous clinical indications for retrieval, variable healing and loading protocols, and complex sample preparation procedures. The available studies also differ in experimental grouping, including implant or abutment system, abutment macrogeometry, surface characteristics, healing time and loading condition, which reduces direct comparability across datasets.

The healing and loading conditions reported in the reviewed studies were determined by the original clinical protocols or retrieval indications, rather than by a controlled longitudinal experimental design. Therefore, the available evidence does not yet allow a systematic time-series analysis of peri-implant collagen maturation.

In addition, several of the key human retrieval and biopsy studies discussed in this review originate from the same or overlapping research groups. This may reflect the limited availability of synchrotron-based peri-implant soft tissue datasets, but it also limits the generalizability of the current evidence and highlights the need for independent replication. Human peri-implant biopsies are difficult to obtain for ethical and practical reasons and retrieved specimens may not fully represent routine clinical conditions.

Fixation, graded dehydration, resin embedding, sectioning, removal of implant or abutment components and subsequent image-processing or segmentation choices may influence tissue morphology, collagen bundle spacing and the apparent continuity of the implant–soft tissue interface. These preparation-related factors may introduce artifacts and should be reported in detail when interpreting SR-PhC-µCT, histological, polarized light microscopy or SEM-based observations. Importantly, the available studies do not establish a causal relationship between specific collagen morphometric parameters and long-term clinical outcomes.

A further limitation is the practical accessibility of SR-PhC-µCT. At present, this technique should be regarded as a highly specialized ex vivo research tool rather than as a clinically available diagnostic modality. Access to synchrotron facilities is limited, beamtime is competitive, and image acquisition requires dedicated instrumentation, optimized beamline conditions and multidisciplinary expertise in sample preparation, X-ray physics, tomographic reconstruction and image analysis. These factors currently restrict the application of SR-PhC-µCT to selected experimental, preclinical and translational research settings.

AI-assisted segmentation may also introduce model-dependent bias. U-Net-based segmentation can distinguish structures according to grayscale, texture, morphology and spatial context, but its performance depends on the size and representativeness of the annotated training dataset, the quality of manual annotations, the balance among semantic classes, tissue maturation stage, collagen density and the prevalence of specific fiber orientations. A model trained predominantly on well-defined mature collagen bundles may perform differently when applied to immature, sparse or highly heterogeneous connective tissue. For this reason, segmentation-derived morphometric parameters require external validation, independent datasets and correlative histological or polarized light microscopy confirmation before they can be considered robust and generalizable biomarkers.

Moreover, claims regarding bacterial leakage, microgap reduction and marginal bone preservation should be interpreted in the context of the specific experimental or clinical conditions under which they were reported. Current evidence does not demonstrate that a given 3D collagen architecture directly prevents bacterial leakage or causally determines marginal bone stability.

Future studies should standardize and fully report acquisition and preprocessing parameters, including beamline or source type, photon energy, beam filtration, exposure time, number of projections, angular range and angular step, sample-to-detector distance, detector characteristics, voxel size, true spatial resolution, reconstruction algorithm and phase-retrieval settings. In addition, the rationale for selecting these parameters should be stated, particularly when imaging collagen-rich tissues where the balance between phase sensitivity, signal-to-noise ratio, spatial resolution and field of view strongly affects segmentation and morphometric analysis. For AI-assisted segmentation, minimum reporting standards should include the size and origin of the annotated dataset, the number of manually annotated slices or volumes, annotator expertise, interobserver variability, training/validation/test partitioning, augmentation strategy, network architecture and hyperparameters, and class-specific segmentation metrics such as the Dice coefficient, intersection over union, precision, recall, sensitivity and specificity. Attention should be paid to whether model accuracy differs between immature and mature connective tissues or between regions with different collagen density and orientation.

SR-PhC-µCT can visualize and quantify collagen-rich architectures based on phase-contrast differences, tissue density gradients and 3D morphology, but it does not directly distinguish collagen types, such as type I and type III. Identification of collagen subtype composition requires correlative methods, including histology, polarized light microscopy, immunohistochemistry, second harmonic generation imaging, spectroscopy or other molecular techniques. Therefore, the microtomographic descriptors discussed in this review should be interpreted as structural parameters of collagen-rich tissue organization rather than as markers of collagen biochemical composition. Moreover, correlative histological and polarized light microscopy validation should also be standardized by reporting section thickness, cutting plane, staining protocol, microscope magnification, polarization configuration, number of analyzed sections and orientation-classification criteria.

Larger multicenter and longitudinal datasets are required to clarify whether morphometric parameters such as collagen volume fraction, anisotropy and connectivity density correlate with clinical outcomes, marginal bone stability, peri-implant disease susceptibility and the performance of specific abutment materials or surface treatments. Until such evidence is available, these parameters should be interpreted as exploratory structural descriptors rather than validated clinical biomarkers.

Computational modeling represents another promising future direction, but it was not investigated in the present review. If microtomographic collagen architecture can be integrated with finite element models and mechanobiological simulations, it may become possible to generate testable predictions on how implant–abutment geometry, surface features, material stiffness and loading protocols influence soft tissue adaptation. However, such applications remain investigational [79,80,81,82,83]. Future finite element studies should explicitly report the material properties assigned to each component, including the elastic modulus and Poisson’s ratio of the implant, abutment, bone and peri-implant soft tissue; the magnitude, direction and point of application of the simulated load; the assumptions regarding contact, friction and bonding at the implant–abutment, bone-implant and soft tissue-abutment interfaces; the boundary conditions; mesh density; convergence criteria; and the experimental or clinical data used for model validation. Until such models are developed and validated, the biomechanical implications of microtomographic collagen architecture should be regarded as hypotheses rather than demonstrated mechanisms.

## 9. Conclusions

Microtomographic imaging, particularly SR-PhC-µCT, provides an advanced framework for characterizing dental implant systems at the material–tissue interface. By enabling three-dimensional visualization of resin-embedded human biopsies, this technique reveals collagen arrangements that cannot be fully understood from conventional two-dimensional sections. The reviewed human retrieval and biopsy studies suggest that peri-implant connective tissue around transmucosal abutments used in Morse-tapered and conometric restorative systems may form a complex interwoven network composed of circumferential and longitudinal collagen bundles.

In conclusion, the available evidence supports the interpretation of Morse-tapered and conometric implant systems as material–biological interfaces in which connection precision, abutment geometry, surface topography and peri-implant tissue microarchitecture are closely interrelated. Current imaging and histological data demonstrate that peri-implant connective tissue may exhibit a complex three-dimensional organization, including circumferential and longitudinal collagen bundles around transmucosal implant components. However, a causal relationship between specific microtomographic collagen parameters and long-term clinical outcomes has not yet been established.

SR-PhC-µCT, particularly when combined with correlative histology and AI-based segmentation, provides an ex vivo approach for quantifying peri-implant soft tissue architecture and generating testable hypotheses on the relationship between implant design and biological response. Nevertheless, the morphometric parameters discussed in this review should currently be regarded as exploratory structural descriptors rather than validated clinical biomarkers.

Because direct comparative three-dimensional studies remain limited, the findings discussed in this review should not be interpreted as evidence that Morse-tapered or conometric systems are uniquely associated with favorable peri-implant collagen organization. Rather, these systems represent clinically relevant models for investigating how connection design, abutment geometry and surface characteristics may interact with peri-implant soft tissue architecture.

Future longitudinal studies, larger human datasets, standardized imaging protocols and independent validation are required before these parameters can be translated into routine clinical decision-making or used to guide implant material design and prosthetic component optimization.

## Figures and Tables

**Table 1:** Representative 3D morphometric parameters of collagen bundles in human peri-implant soft tissue specimens around Morse-tapered abutments, adapted from Iezzi et al. [10]. Values are reported as mean ± standard deviation from three human retrieved implants with cone Morse implant–abutment connections, as reported in the original study. No new statistical comparison was performed for the present review; the table is intended to provide representative descriptive morphometric values rather than newly generated inferential statistics. The implants were inserted equicrestally, immediately loaded and retrieved after 4–6 weeks. The analyzed subvolumes were approximately 19 × 10^6^ µm^3^ and collectively mapped the biopsy. Transverse/circular collagen bundles were analyzed as a single shell because of the limited biopsy thickness in the transverse plane, with a mean thickness of 300 µm. Longitudinal collagen bundles were analyzed in two shells, inner and outer, each with a mean thickness of 250 µm.

Parameter	Transversal/Circular CB	Longitudinal CB-Inner	Longitudinal CB-Outer
Vol.%	35 ± 25	38 ± 6	51 ± 10
Th (µm)	33 ± 15	36 ± 6	47 ± 11
Nr (mm^−1^)	9 ± 2	10 ± 1	10 ± 2
Sp (µm)	78 ± 38	64 ± 10	52 ± 16
DA	0.70 ± 0.20	0.61 ± 0.04	0.67 ± 0.05
Conn.D (×10^−6^ px^−3^)	10.4 ± 5.3	13.4 ± 3.3	13.3 ± 4.4
FD	2.35 ± 0.07	2.39 ± 0.02	2.42 ± 0.07

Note: Vol.%: collagen volume fraction; CB: collagen bundles; Th: mean collagen bundle thickness; Nr: specific number of collagen bundles; Sp: mean collagen bundle spacing; DA: degree of anisotropy; Conn.D: connectivity density; FD: fractal dimension. Thickness and spacing are expressed in µm; number is expressed in mm^−1^; DA and FD are dimensionless. Connectivity density is reported in the pixel-based unit used in the original study. Because pixel-based connectivity density depends on voxel size, image resolution, segmentation strategy and region-of-interest definition, it should be interpreted as a dataset-specific structural descriptor and should not be directly compared across studies unless calibrated physical units and standardized workflows are used.

**Table 2:** Examples of investigated samples categorized by healing duration and loading condition, adapted from Riberti et al. [73].

Sample	Time Before Retrieval	Immediate Loading	Region
Case 1	6 weeks	Yes	Not specified
Case 2	10 years	No	Not specified
Case 3	6 weeks	No	Not specified
Case 4	8 weeks	No	Right posterior
Case 5	8 weeks	No	Left posterior

## Data Availability

No new data were created or analyzed in this study. Data sharing is not applicable to this article.

## Supplementary Materials

The following supporting information can be downloaded at: https://www.mdpi.com/article/10.3390/ma19153272/s1, Table S1: Summary of key studies included in the focused narrative review.

## Abbreviations

The following abbreviations are used in this manuscript:

3D	Three-dimensional
µCT	micro-computed tomography
SR-PhC-µCT	synchrotron radiation-based phase-contrast micro-computed tomography
Vol%	collagen volume fraction
Th	mean collagen bundle thickness
Sp	mean collagen bundle spacing
Nr	specific number of collagen bundles
Conn.D	connectivity density
DA	degree of anisotropy
FD	fractal dimension
CB	collagen bundles
AI	artificial intelligence
IoU	intersection over union
ROI	region of interest
