# Supplementary material for: Microtomographic Characterization of Morse-Tapered Implant Systems as Material–Biological Interfaces: Implications for Peri-Implant Soft Tissue Stability
Source: Materials (Basel). 2026 Aug 3;19(15):3272. doi: 10.3390/ma19153272 (PMC13467643; doi:10.3390/ma19153272)
Supplement: Supplementary file 1 [file materials-19-03272-s001.zip › materials-4426726-supplementary.pdf]

Table S1. Summary of key studies included in the focused narrative review. The table has been revised to report, whenever available from the original publications, sample size, grouping criteria, implant/abutment system, surface characteristics, healing/loading conditions, imaging/acquisition parameters, analysis workflow, main outcomes and limitations relevant to reproducibility.

Study	Study type / role in review	Sample size / model	Experimental grouping, implant/abutment system and surface characteristics	Healing/loading or sample condition	Imaging, acquisition and analysis details	Main outcomes relevant to the review	Key limitations / reproducibility notes
**Iezzi et al. [10]**	Human retrieval / ex vivo proof-of-concept study focused on 3D peri-implant connective tissue organization.	3 human retrieved implants with surrounding peri-implant soft tissues; cone Morse implant-abutment connection.	No parallel experimental groups. Implants inserted equicrestally, immediately loaded and retrieved after 4-6 weeks; specimens selected from archived human retrieval material.	Immediate functional loading; early healing/retrieval after 4-6 weeks. Implants were stable and well osseointegrated; no inflammation reported before retrieval.	Histology, polarized light microscopy and SR-PhC-microCT. SYRMEP beamline (Elettra); average energy 19 keV; exposure 0.2 s/projection; 180° angular range; sample-detector distance 100 mm; isotropic pixel size 890 nm; Paganin phase retrieval with δ/β= 100. Several subvolumes of approximately 19 x 10^6 µm^3 mapped the biopsy. Morphometry with ImageJ/BoneJ after preprocessing/thresholding.	Identified interwoven circumferential/transverse and longitudinal collagen bundles around the abutment, sparse vascular structures and no clear radial insertion into the implant surface. Provided representative descriptors including collagen volume fraction, bundle thickness, spacing, degree of anisotropy, connectivity density and fractal dimension.	Very small sample size; early healing interval; no control group with other connection designs; archived retrieval conditions not representative of routine long-term clinical function; morphometric units and thresholds are dataset-specific.
**Canullo et al. [66]**	Human observational study evaluating abutment macro- and micro-geometry with histology, polarized light and SR-PhC-microCT.	30 patients/30 peri-implant soft tissue samples planned: 10 DIV-MAC, 10 CONV-MAC and 10 CONV-UTM. For PhC-microCT, 22 abutments were finally analyzed after 8 exclusions during processing/cutting: 8 DIV-MAC, 7 CONV-MAC, 7 CONV-UTM.	Three healing abutment designs in Morse cone implant systems: divergent machined abutment (DIV-MAC), convergent machined abutment (CONV-MAC), and convergent abutment with ultrathin threaded microsurface (CONV-UTM). MAC surface: circular micro-threads <2 micrometers, Sa 0.2, Ra 0.11; UTM: triangular threading, pitch >50 micrometers, Sa 0.6, Ra 0.62.	One-stage protocol; healing abutments placed at implant insertion; peri-implant soft tissue harvested after 3 months of healing. Loading was not the primary experimental variable.	Histology, polarized light microscopy and SR-PhC-microCT. SYRMEP beamline (Elettra); 2 GeV, 300 mA; 1 mm silicon filter; average energy 19.3 keV; exposure 0.4 s/projection; 3600 projections over 360°; sample-detector distance 100 mm; pixel size 890 nm; Paganin δ/β= 100. Subvolumes 540 x 270 x 270 µm^3, positioned close to the mucosa/abutment interface and stacked longitudinally. Morphometry with BoneJ/ImageJ.	Convergent abutments showed greater connective tissue vertical dimension than divergent abutments. CONV-UTM showed significantly higher collagen specific volume and higher connectivity density than DIV-MAC in selected comparisons. Results support a potential influence of abutment macrogeometry and surface microtopography on peri-implant connective tissue architecture.	Observational human design; moderate final sample size after exclusions; healing abutments rather than definitive prosthetic components; three-month healing only; some technical exclusions during sample processing; no longitudinal clinical outcome correlation.
**Riberti et al. [73]**	Demonstrative human peri-implant soft tissue imaging study comparing thresholding and U-Net-based semantic segmentation.	Peri-implant soft tissues from 4 patients, represented by 5 samples/biopsies. Four subvolumes were extracted from each sample.	Samples represented different healing/loading conditions rather than randomized material groups: immediately loaded short-term sample, non-immediately loaded long-term sample, and non-loaded short-term samples from posterior jaw sites. Implant/abutment system details were archival and not used as primary grouping variables.	Case 1: 6 weeks, immediately loaded; Case 2: long-term, not immediately loaded; Case 3: 6 weeks, not loaded; Case 4 and Case 5: 8 weeks, not loaded, right/left posterior sites.	SR-PhC-microCT at SYRMEP beamline (Elettra); 1800 projections; 0.2 s/projection; 180° angular range; peak energy approximately 17 keV; sample-detector distance 100 mm; pixel size 890 nm; Paganin phase retrieval, δ/β= 100. Dragonfly 2022.1 for U-Net semantic segmentation; Fiji/BoneJ for morphometry; Otsu thresholding used as comparative method. Four volumes of 600 x 300 x 300 pixels^3 (~0.04 mm^3) selected per sample. U-Net trained on 50 manually segmented slices, three classes, 90/10 training/validation split, 300 epochs, categorical cross-entropy, Adadelta, categorical accuracy.	U-Net segmentation allowed transverse/circular and longitudinal collagen bundles to be quantified separately, unlike grayscale thresholding alone. In most mature samples, transverse bundles showed higher volume fraction and connectivity density than longitudinal bundles; immature Case 4/5 tissues showed greater variability. Training/validation accuracy was reported, but Dice/IoU were not provided.	Small heterogeneous dataset; limited manual training set; no external validation dataset; no Dice coefficient or IoU reported; potential model dependence on tissue maturity and fiber orientation; results should be considered methodological proof-of-concept.
**Riberti et al. [74]**	Human clinical case report/retrieval study combining laboratory micro-CT, SR-PhC-microCT, histology, polarized light microscopy and AI-assisted analysis.	One retrieved implant with peri-implant soft tissue from a 61-year-old female patient; two additional identical commercial conometric implant-abutment-cap assemblies were analyzed by laboratory micro-CT for interface characterization.	Conometric abutment-prosthetic cap system. Retrieved implant: titanium implant, 3.3 x 8 mm, with straight titanium conometric abutment and PEEK conometric cap. Two commercial assemblies were pre-assembled with abutment and cap for gap evaluation.	Implant placed 1 mm subcrestally in the left maxillary posterior region; retrieved after 45 days because of prosthetic mispositioning/prosthetic needs, not biological failure. Wound healing remained undisturbed before retrieval.	Laboratory micro-CT of 2 commercial assemblies: Bruker Skyscan 1174; 50 kV, 800 microA; pixel size 6.5 micrometers; 1.5 mm Al filter; exposure 17.5 s; 180° scan, 0.3°step. SR-PhC-microCT of peri-implant tissue: SYRMEP beamline; 1800 projections; 0.2 s/projection; 180° scan; peak energy 17 keV; sample-detector distance 100 mm; pixel size 890 nm; δ/β= 100; Paganin phase retrieval. Dragonfly and BoneJ/Fiji for AI-assisted morphometry; histology and polarized light microscopy for correlation.	Laboratory micro-CT showed no detectable macro- or microgap in the analyzed conometric abutment-prosthetic cap assemblies. SR-PhC-microCT and AI-assisted analysis showed mature, collagen-rich peri-implant soft tissue with intertwined longitudinal and transverse bundles; transverse bundles had higher number/connectivity than longitudinal bundles.	Single clinical retrieval; two additional implant assemblies assessed only ex vivo for connection gaps; short healing period; retrieval due to prosthetic mispositioning; no comparative group; conclusions are exploratory and not generalizable to all conometric systems.
**Furlani et al. [9]**	Perspective / methodological article on SR-PhC-microCT and deep learning for collagenous tissues.	Literature-based perspective with demonstrative examples; not an original peri-implant experimental cohort.	Not applicable as experimental grouping. Discusses collagen-rich tissues and includes peri-implant soft tissue examples as a methodological use case.	Not applicable.	Discusses SR-PhC-microCT for collagenous tissues and the rationale for deep learning-based semantic segmentation when grayscale thresholding cannot distinguish structures with similar density but different orientation. Highlights U-Net as a suitable architecture for biomedical image segmentation.	Supports the methodological rationale for using AI to distinguish collagen bundles according to orientation and not only physical density; emphasizes the need for sample size, validation and rigorous statistics in future work.	Perspective article, not a systematic review or experimental validation study; no new standardized segmentation metrics such as Dice or IoU for peri-implant datasets.

Abbreviations: SR-PhC-microCT, synchrotron radiation-based phase-contrast micro-computed tomography; MAC, machined; UTM, ultrathin threaded microsurface; DIV, divergent; CONV, convergent; DA, degree of anisotropy; Conn.D, connectivity density; IoU, intersection over union; N/A, not applicable.

Note: This supplementary table is intended to increase transparency in a narrative review. It does not represent a de novo systematic extraction or meta-analysis. Parameters reported as "not applicable" refer to review articles or studies in which the requested experimental variable was not part of the original design. When quantitative validation metrics such as Dice coefficient or IoU were not reported in the original segmentation studies, this is indicated as a limitation rather than inferred.
